# Effects of virtual reality rehabilitation training on gait and balance in patients with Parkinson's disease: A systematic review

**DOI:** 10.1371/journal.pone.0224819

**Published:** 2019-11-07

**Authors:** Cheng Lei, Kejimu Sunzi, Fengling Dai, Xiaoqin Liu, Yanfen Wang, Baolu Zhang, Lin He, Mei Ju

**Affiliations:** 1 Department of Nursing, People’s Hospital of Deyang, Deyang, Sichuan, China; 2 School of Nursing, Southwest Medical University, Luzhou, Sichuan, China; 3 Faculty of Nursing, Chiang Mai University, Chiang Mai, Thailand; University rehabilitation institute, SLOVENIA

## Abstract

**Objective:**

In recent years, virtual reality (VR) has been tested as a therapeutic tool in neurorehabilitation research. However, the impact effectiveness of VR technology on for Parkinson’s Disease (PD) patients is still remains controversial unclear. In order to provide a more scientific basis for rehabilitation of PD patients’ modality, we conducted a systematic review of VR rehabilitation training for PD patients and focused on the improvement of gait and balance.

**Methods:**

An comprehensive search was conducted using the following databases: PubMed, Web of Science, Cochrane Library, CINHAL, Embase and CNKI (China National Knowledge Infrastructure).Articles published before 30 December 2018 and of a randomized controlled trial design to study the effects of VR for patients with PD were included. The study data were pooled and a meta-analysis was completed. This systematic review was conducted in accordance with the PRISMA guideline statement and was registered in the PROSPERO database (CRD42018110264).

**Results:**

A total of sixteen articles involving 555 participants with PD were included in our analysis. VR rehabilitation training performed better than conventional or traditional rehabilitation training in three aspects: step and stride length (SMD = 0.72, 95%CI = 0.40,1.04, Z = 4.38, P<0.01), balance function (SMD = 0.22, 95%CI = 0.01,0.42, Z = 2.09, P = 0.037), and mobility(MD = -1.95, 95%CI = -2.81,-1.08, Z = 4.41, P<0.01). There was no effect on the dynamic gait index (SMD = -0.15, 95%CI = -0.50,0.19, Z = 0.86, P = 0.387), and gait speed (SMD = 0.19, 95%CI = -0.03,0.40, Z = 1.71, P = 0.088).As for the secondary outcomes, compared with the control group, VR rehabilitation training demonstrated more significant effects on the improvement of quality of life (SMD = -0.47, 95%CI = -0.73,-0.22, Z = 3.64, P<0.01), level of confidence (SMD = -0.73, 95%CI = -1.43,-0.03, Z = 2.05, P = 0.040), and neuropsychiatric symptoms (SMD = -0.96, 95%CI = -1.27,-0.65, Z = 6.07, P<0.01), while it may have similar effects on global motor function (SMD = -0.50, 95%CI = -1.48,0.48, Z = 0.99, P = 0.32), activities of daily living (SMD = 0.25, 95%CI = -0.14,0.64, Z = 1.24, P = 0.216), and cognitive function (SMD = 0.21, 95%CI = -0.28,0.69, Z = 0.84, P = 0.399).During the included interventions, four patients developed mild dizziness and one patient developed severe dizziness and vomiting.

**Conclusions:**

According to the results of this study, we found that VR rehabilitation training can not only achieve the same effect as conventional rehabilitation training. Moreover, it has better performance on gait and balance in patients with PD. Taken together, when the effect of traditional rehabilitation training on gait and balance of PD patients is not good enough, we believe that VR rehabilitation training can at least be used as an alternative therapy. More rigorous design of large-sample, multicenter randomized controlled trials are needed to provide a stronger evidence-based basis for verifying its potential advantages.

## Introduction

Parkinson's disease (PD) is a common neurodegenerative disease with progressive development [1]. The pathological changes of the disease are degeneration of the substantia nigra and striatum pathway [2]. In general, the incidence of PD in people before the age of 60 is 0.13% to 1.6%, but with the increase of age, the incidence of PD in people aged 80 to 84 can be as high as 9% [3].The clinical manifestations are motor symptoms such as bradykinesia, dystonia, tremor and postural balance disorder, and non-motor symptoms such as cognitive decline and depression [4]. Currently, drug therapy is the preferred treatment for PD, but it is effective only for the first years after onset and some symptoms do not respond at all to drug treatment [5, 6].Deep brain stimulation is one of the current treatments for primary PD where electrodes are implanted in the brain to stimulate the targeted area and improve the related symptoms of PD patients. However, improper intraoperative electrode positioning or stimulation parameters may not only affect the therapeutic effect, but also stimulate the peripheral nerve conduction bundle, causing various types of motor, sensory symptoms and other adverse reactions [7]. It has been reported that long-term rehabilitation training can improve the motor ability and cognitive outcome of patients with PD [8, 9]. However, in actual clinical practice, long-term rehabilitation training has high requirements for the ability of rehabilitation therapists, the financial situation of patients, training places, and the safety of patients. As a result, it is difficult for people with PD to obtain and maintain long-term regular training.

In recent years, virtual reality (VR) as a therapeutic tool has become a new topic in neurorehabilitation research [10]. From the perspective of kinematics learning, VR provides a possibility for high-intensity, task-oriented and multi-sensory feedback training, which can promote patients' visual, auditory and tactile input, and increase their interest in the rehabilitation process by letting patients experience immersion or non-immersion virtual environment, so that patients' treatment compliance is effectively improved [11, 12]. Previous studies have confirmed that VR technology plays an active role in stroke [13], cognitive function and quality of life in the elderly [14]. It has also been shown that VR can improve the balance function and daily life activities of patients with PD [15]. However, a systematic review from Cochrane Library showed that VR rehabilitation training had a positive impact only on stride speed and stride length of patients with PD compared to traditional physical training [16].The small number of studies and limited sample size for testing of VR rehabilitation training have yielded inconsistent results. Therefore, the impact of VR technology on PD patients is still controversial.

In order to provide a more scientific basis for the rehabilitation of PD patients, we conducted a systematic review and meta-analysis of VR rehabilitation training for PD patients.

## Methods

### Data sources and search strategy

This meta-analysis was executed following the (Preferred Reporting Items for Systematic Reviews and Meta-Analyses) PRISMA guidelines(**S1 Checklist**) and was registered in the PROSPERO database (CRD42018110264).

We conducted an electronic search through the following databases: PubMed, Web of Science, Cochrane Library, CINHAL, Embase, and CNKI (China National Knowledge Infrastructure).All databases were searched from the establishment of the database to 30 December2018. The main terms including "Parkinson’s disease,""Parkinsonian,""pd" and "virtual reality,""VR,""kinect,""Wii,""X-box" and their related synonyms were taken into consideration. The detail strategy is presented in S1 Appendix.

### Research question and study selection

Our research question was based following on the PICOS principle (Population, Intervention, Comparison, Outcome measures and Study design). We included studies involving participants who were clinically diagnosed with PD, without any limitations on gender, age, disease duration or severity. The experimental group in each study was treated with exercise and motor rehabilitation training based on VR technology. We define VR intervention as "rehabilitating training based on a computer simulation: users appear in the virtual environment in real time through a variety of sensory modes and interact with images or virtual objects.". This includes interactive motion-sensing training or commercial video training games (such as Nintendo Wii Fit, x-box 360, etc.). There were no limits on the frequency or duration of VR rehabilitation training. The control group received routine physical rehabilitation therapy or standard of care such as health education, rehabilitation care, physiotherapist supervised training or any other non-VR exercise intervention. All studies were designed as randomized controlled trials, other methodological designs were excluded.

### Types of outcome measures

The primary outcomes we collected included three aspects: (1) Gait. Dynamic Gait Index (DGI) [17], Six or Ten Minute Walk Test (6MWT or 10MWT) [18, 19] were used to evaluated gait. Gait speed and step and stride length were calculated using the results of scale measurements. (2) Balance function. Measures of balance function were completed with Center of Pressure behavior (CoP) [20], Berg Balance Scale (BBS) [21] and Dynamic Balance Performance (DBP) [22]. (3) Mobility. Timed Up and Go Test (TUGT) [23] were used to measure changes in subjects' mobility.

Our collection of secondary outcomes includes the following: (1) Global motor function. The Unified Parkinson’s Disease Rating Scale partⅢ[21] was used to measure global motor function changes. (2) Activities of daily living (ADL). UPDRS part II [21] and the Barthel Index [24] were employed to measure ADL. (3) Quality of life. Quality of life was determined by the 39-Item Parkinson’s Disease Questionnaire (PDQ-39) [17] and the World Health Organization Quality of Life for Older Persons (WHOQOL-OLD) [19]. (4) Perceived confidence in balance. The Falls Efficacy Scale (FES) [25] and Activities-specific Balance Confidence Scale (ABC) [26] were used to measure patient’s level of confidence in doing specific activities that could affect balance and cause falls. (5) Neuropsychiatric symptoms. Beck Anxiety Inventory (BAI) [19], Beck Depression Inventory (BDI) [24], 15-item Geriatric Depression Scale (GDS-15) [27], and Hamilton Depression Scale (HAMD) [20]were used to record neuropsychiatric symptoms changes in subjects. (6) Cognitive function. Cognitive function was measured by the Montreal Cognitive Assessment (MCA) [21], Mini-Mental State Examination (MMSE)[26], and Digit Span forward (DSF)[19]. (7) Adverse events. The number and type of adverse events reported in each study were collected.

The outcome measures used in this study were extracted from the included literatures.

### Data extraction and quality assessment (risk of bias)

The titles and abstracts of the retrieved literature, were initially screened by two independent reviewers (L.C and SZ.K), and duplicates were eliminated by Endnote. After that, the full text of the selected study was analyzed to determine whether the study met the inclusion and exclusion criteria. After identifying suitable articles for inclusion in this study, data extraction and quality assessment (risk of bias) were carried out independently by the above evaluators. The extraction contents include research design, research location, inclusion and exclusion criteria, research objects, intervention measures, control measures, outcome index evaluation time and so on. If the data of the study report is incomplete, the original author was contacted to obtain it. If relevant data were not able to be obtained, the study was excluded. Quality assessment was done using Cochrane Handbook for Systematic Reviews of Interventions Version 5.1.0.The criteria included: (1) random sequence generation; (2) allocation concealment; (3) blinding of outcome assessment; (4) incomplete outcome data; (5) selective outcome reporting. Each criterion was judged as “low risk of bias”, “high risk of bias” or “unclear risk of bias“. Because of the particularity of VR intervention, blind method was not applicable to subjects and therapists, so it was not included in the “risk of bias” assessment. All disagreements were resolved through third researcher (D.FL).

### Data synthesis and meta-analysis

The primary outcomes and secondary outcomes concerned in this paper are continuous variables. If possible, we pooled the change values of each indicator before and after intervention. Mean differences (MD) or standardized mean difference (SMD) and its 95% confidence interval (CI) were the results of meta-analysis. We visually assessed heterogeneity through forest plots and *I*^*2*^statistic made funnel plot to judge publication bias. When *P*<0.05 or *I*^*2*^≥50%, it was considered that there was substantial heterogeneity, and a random effect model was selected, otherwise a fixed effect model was selected for analysis. For all the result variables, two-tailed *P*<0.05 was considered to be statistically significant. Stata 12.0 (StataCorp. College Station, Texas) was used to pool the effects and construct the plots.

## Results

### Study selection and methodological quality assessment (risk of bias)

**Fig 1:**
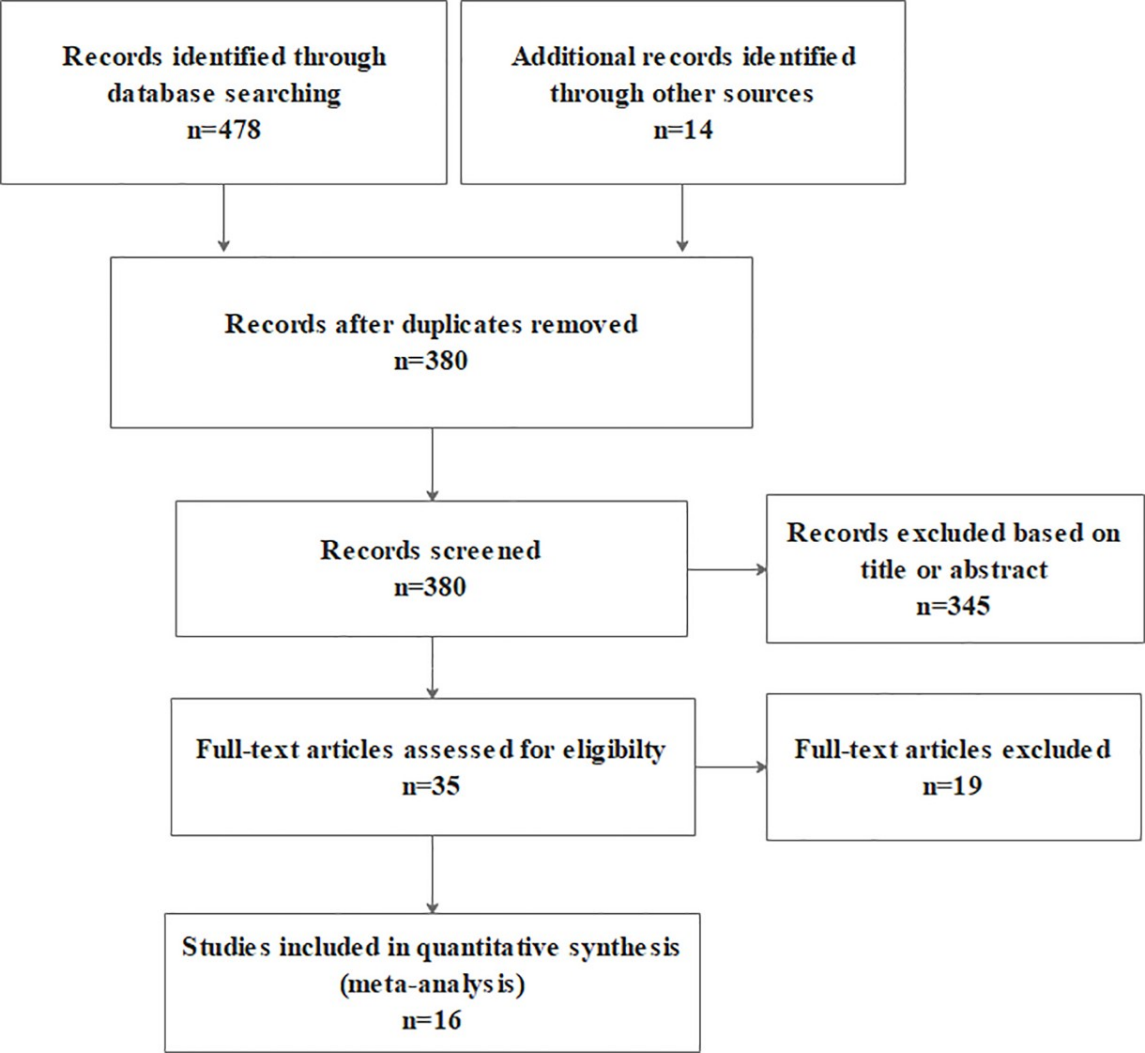
Flowchart of trial selection.

A total of sixteen articles [17–32] involving 555 participants with PD were included in the systematic review. The flowchart of study selection was detailed in Fig 1. Random sequence allocation was sufficient in fifteen (94%)articles[17–28, 30–32]. Information on concealment of treatment allocation was reported in fifteen (94%) articles[17–27, 29–32].Eight (50%) trials [17, 18, 21, 25, 26, 28, 31, 32] reported adequate blinding of the outcome assessor. As mentioned above, due to the particularity of VR intervention, the blindness of participants and personnel did not apply, so we did not evaluate this item. Eleven (69%) articles[17, 18, 20–25, 27, 28, 32]clearly provided complete data, while there were high risk of attrition bias in two (12%) articles[29, 30].There were thirteen (81%) articles[17–22, 24, 26, 28–32]was considered to be at unclear risk of bias about selective reporting. All articles included in our study were assessed with varying degrees of risks bias except van de Heuvel [25], which indicated that the overall quality of the articles included were at a medium level. The methodological quality of the included articles can be referred to in Table 1.

**Table 1:** Authors' judgments about each risk of bias item for each included study.

Studies/Items	Random sequence generation	Allocation concealment	Blinding of outcome assessment	Incomplete outcome data	Selective outcome reporting
**Alves 2018**	Low	Low	Unclear	Unclear	Unclear
**Chen 2017**	Low	Low	Unclear	Low	Unclear
**Ferraz 2018**	Low	Low	Unclear	Low	Low
**Gandolfi 2017**	Low	Unclear	Low	Low	Unclear
**van de Heuvel 2014**	Low	Low	Low	Low	Low
**Laio 2014**	Low	Low	Unclear	Low	Unclear
**Lee 2015**	Low	Low	Unclear	Low	Unclear
**Lin 2016**	Unclear	Low	Unclear	High	Unclear
**Özgönenel 2016**	Low	Low	Unclear	Unclear	Unclear
**Pedreira 2013**	Low	Low	Low	High	Unclear
**Pompeu 2012**	Low	Low	Low	Low	Unclear
**Ribas 2017**	Low	Low	Low	Low	Unclear
**Shih 2016**	Low	Low	Unclear	Low	Low
**Yang 2015**	Low	Low	Low	Low	Unclear
**Shen 2014**	Low	Low	Low	Unclear	Unclear
**Yen 2011**	Low	Low	Low	Low	Unclear

### Study design and population characteristics

16 There were references were finally included which and all were published from 2011 to 2018.All the experimental groups included in our study adopted VR-based interventions, such as balance training, sports games, visual feedback training and gait training. The intervening time ranged from 30 minutes to 1 hour, and the intervening period ranged from 4 to 12 weeks. While patients in the control group received either traditional or conventional rehabilitation training. Additional information about the interventions is available in Table 2.

**Table 2:** Main characteristics of included studies.

study	Age Gender(M/F)	Sample size	EG	CG	Hoehn-Yahr scale	Dosage	Outcomes	Adverse events
Pompeu 2012	67.4±8.1 17/15 53.1%/46.9%	32	Wii-based motor training	Traditional balance exercise	Stage 1 and 2	60 minutes/day 2 days/week 7 weeks	BBS,UPDRS,MCA	Number of adverse events
Ribas 2017	60.95±9.11 8/12 40.0%/60.0%	20	Nintendo Wii ^TM^ fit game	Traditional balance exercise	EG:1.25(1.0–2.0) CG:1.5(1.0–2.0)	30 minutes/day 2 days/week 12 weeks	BBS,ADL,6MWT	-
Shih 2016	68.15±9.58 16/4 80.0%/20.0%	20	Balance-based exergaming game	Conventional balance training	EG:1.6±0.84 CG_1_:1.4±0.52	50 minutes/day 2 days/week 8 weeks	BBS,TUGT	-
Yang 2015	74.17±7.25 14/9 60.9%/39.1%	23	Customized balance board therapy	Conventional balance training	EG:3.0(3.0–3.0) CG:3.0(3.0–3.0)	50 minutes/day 2 days/week 6 weeks	BBS,TUGT,UPDRS,DGI,PDQ-39	-
Alves 2018	61.07±10.74 25/2 92.6%/7.4%	27	EG_1_:Nintendo Wii ^TM^ fit game EG_2_:Xbox 360 motion games	Conventional balance training	EG_1_:1.89±0.92 EG_2_:1.56±0.72 CG:1.78±0.83	45–60 minutes/day 3–5 days/week 5 weeks	TUGT,10MWT,BAI,DSF,WHOQOL-OLD	-
Chen 2017	63.37±5.7 26/20 56.5%/43.5%	46	BioFlex-FP	Conventional rehabilitation training	EG:2.57±0.50 CG_1_:2.52±0.51	50 minutes/day 5 days/week 6 weeks	BBS, TUGT, CoP, HAMD, UPDRS	-
Ferraz 2018	66.73±12.45 37/25 59.7%/40.3%	62	Xbox 360 game with Kinect	CG_1_:Conventional functional training CG_2_:Bicycle exercise training	EG:2.5(2.5–3.0) CG_1_:2.5(2.0–3.0) CG_2_:2.5(2.0–2.5)	50 minutes/day 3 days/week 8 weeks	PDQ-39,6MWT,10MWT,GDS-15	-
Gandolfi 2017	68.65±8.4 51/25 67.1%/32.9%	76	TeleWii balance training	Conventional balance training	EG:2.5(2.5–2.5) CG:2.5(2.5–3.0)	50 minutes/day 3 days/week 7 weeks	BBS,DGI,ABC,10MWT	Number of falls
van de Heuvel 2014	67.51±8.12 20/13 60.6%/39.4%	33	Visual feedback training	Conventional balance training	EG:2.5(2.0–3.0) CG:2.5(2.0–2.5)	60 minutes/day 7days/week 5 weeks	BBS,UPDRS,PDQ-39,FES	Number of falls
Laio 2014	65.67±7.39 17/19 47.2%/52.8%	36	VR-based Wii Fit exercise	CG_1_:Fall-prevention education CG_2:_Traditional balance exercise	EG:2.0±0.7 CG_1_:1.9±0.8 CG_2_:2.0±0.8	45 minutes/day 2 days/week 6 weeks	TUGT,PDQ-39,DBP,SOT,FES	Number of adverse events
Lee 2015	69.25±3.15 10/10 50%/50%	20	Nintendo Wii ^TM^ fit game	Conventional balance training	-	45 minutes/day 5 days/week 6 weeks	BBS,ADL,BDI	-
Lin 2016	61.72±7.29 22/11 66.7%/33.3%	33	Xbox 360 motion games	Conventional balance training	EG:2.7±0.9 CG:2.9±0.7	30 minutes/day 5 days/week 4 weeks	BBS,TUGT,ADL	Number of adverse events
Özgönenel 2016	65 (46–78) 22/11 66.7%/33.3%	33	Xbox video game console	Conventional rehabilitation program	Stage 1 = 7 Stage 2 = 16 Stage 3 = 10	60 minutes/day 3 days/week 5 weeks	BBS,TUGT,UPDRS	-
Pedreira 2013	63.57±8.61 22/9 71.0%/29.0%	31	Nintendo Wii ^TM^ fit game	Traditional balance exercise	EG:2.5±0.6 CG_1_:2.4±0.7	40 minutes/day 3 days/week 4 weeks	PDQ-39	-
Yen 2011	70.3±6.6 24/4 85.7%/14.3%	28	Customized balance board therapy	Conventional balance training	EG:2.6±0.5 CG_1_:2.4±0.5	30 minutes/day 2 days/week 12 weeks	SOT	Number of falls
Shen 2014	64.2±8.4 22/13 62.9%/37.1%	35	Computerized Balance and gait training system	Conventional strength training	EG:2.4±0.5 CG_1_:2.5±0.5	60 minutes/day 3 days/week 6 weeks	ABC, Gait velocity, Stride length, MMSE	Number Of falls

M:Male; F:Female;EG:experimental group; CG: control group; TUGT: Timed Up and Go Test; BBS: Berg Balance Scale; ADL: Activities of Daily Living;PDQ-39:39-Question Parkinson’s Disease Questionnaire; UPDRS: Unified Parkinson's Disease Rating Scale; DGI: Dynamic Gait Index WHOQOL-OLD: World Health Organization Quality of Life-Old; BAI: Beck Anxiety Index; BDI: Beck Depression Index; HAMD: Hamilton Depression Scale;GDS-15: 15-item Geriatric Depression Scale;6MWT & 10MWT:Six & Ten Minute Walk Test; MCA: Montreal Cognitive Assessment; MMSE: Mini-Mental State Examination; DSF:Digit Span forward; ABC: Activities-specific Balance Confidence Scale; FES: Falls Efficacy Scale; DBP:Dynamic Balance Performance; SOT:Sensory Organization Test; ABC:Activities-specific Balance Confidence Scale; CoP:Center of Pressure.

### Results of primary outcomes

#### Gait

**Fig 2:**
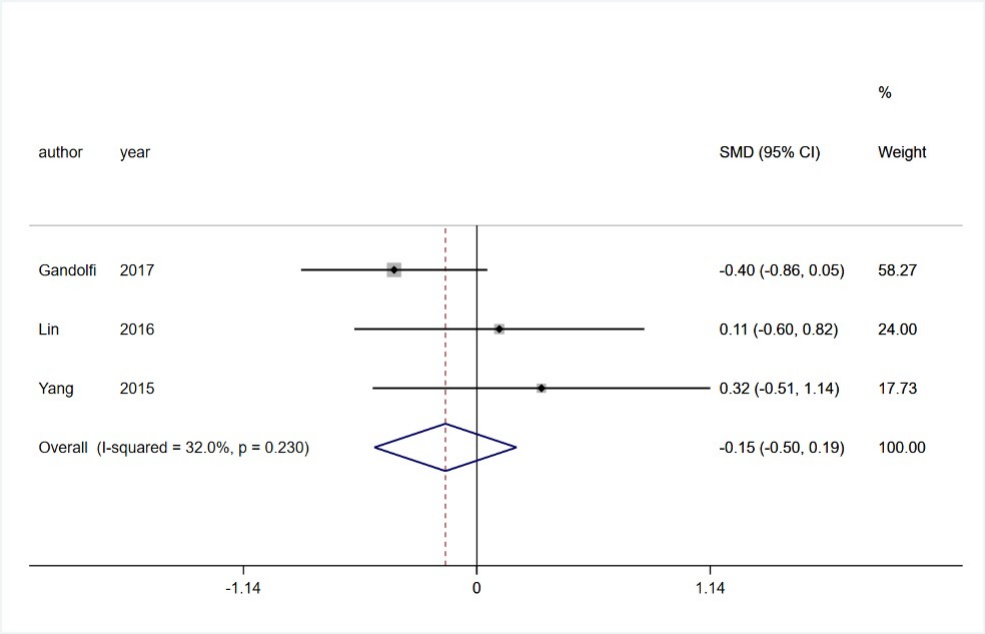
Forest plot of DGI.

**Fig 3:**
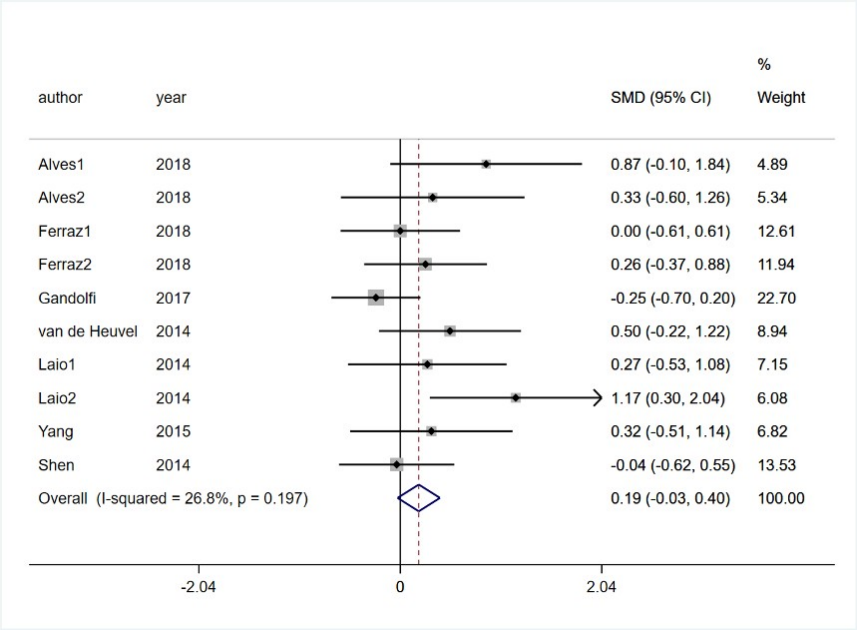
Forest plot of Gait speed.

**Fig 4:**
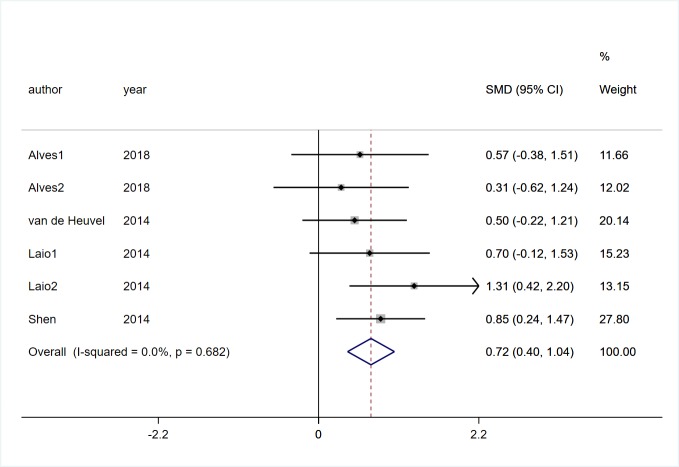
Forest plot of Step and stride length.

Three studies [17, 28, 29] involving 130 participants with PD reported the effects of VR technology on dynamic gait index (DGI). Low heterogeneity was observed (*I*^*2*^ = 32%, *P* = 0.23). Pooled SMD showed that There was no significant difference between two groups (SMD = -0.15, 95%CI = -0.50, 0.19, Z = 0.86, P = 0.387, Fig 2).Seven studies [17, 19, 22, 25–28] involving 347 participants with PD reported the effects of VR technology on gait speed. Low heterogeneity was observed (*I*^*2*^ = 26.8%, *P* = 0.197). Pooled SMD showed that there was no significant difference between two groups (SMD = 0.19, 95%CI = -0.03, 0.40, Z = 1.71, *P* = 0.088, Fig 3). Four trials [19, 22, 25, 26] involving 166 participants with PD recorded the effects of VR technology on step and stride length, and there was no statistical heterogeneity was observed (*I*^*2*^ = 0%, *P* = 0.682). Pooled SMD showed that VR rehabilitation training had a significant effect on step and stride length (SMD = 0.72, 95%CI = 0.40, 1.04, Z = 4.38, *P*<0.01, Fig 4).

#### Balance function

**Fig 5:**
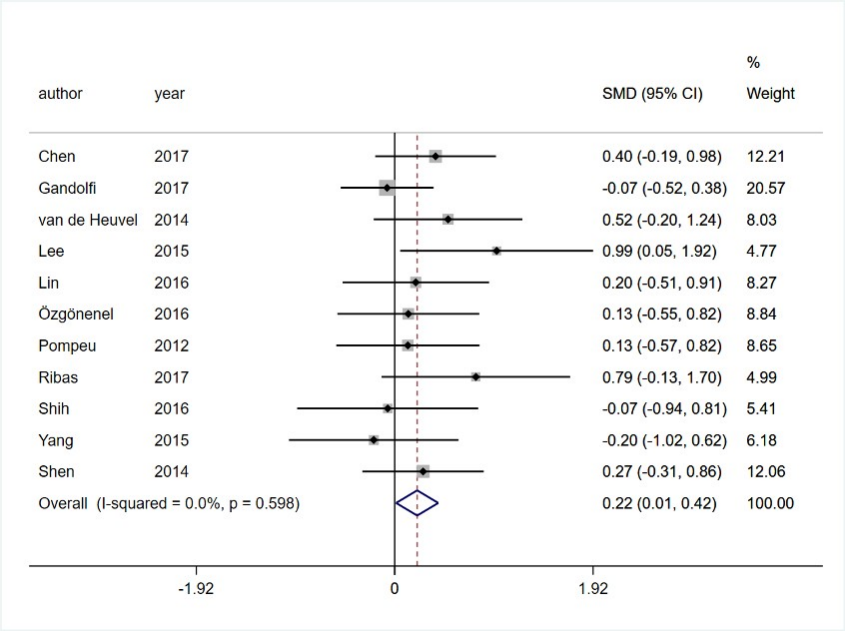
Forest plot of Balance function.

Eleven studies [17, 18, 20, 21, 23–26, 28–30] involving 360 participants with PD provided complete data on balance function. Pooled SMD showed that VR rehabilitation training had a significant effect on balance function (SMD = 0.22, 95%CI = 0.01, 0.42, Z = 2.09, *P* = 0.037, Fig 5). The meta-analysis showed low statistical heterogeneity (*I*^*2*^ = 0%, *P* = 0.598).

#### Mobility

**Fig 6:**
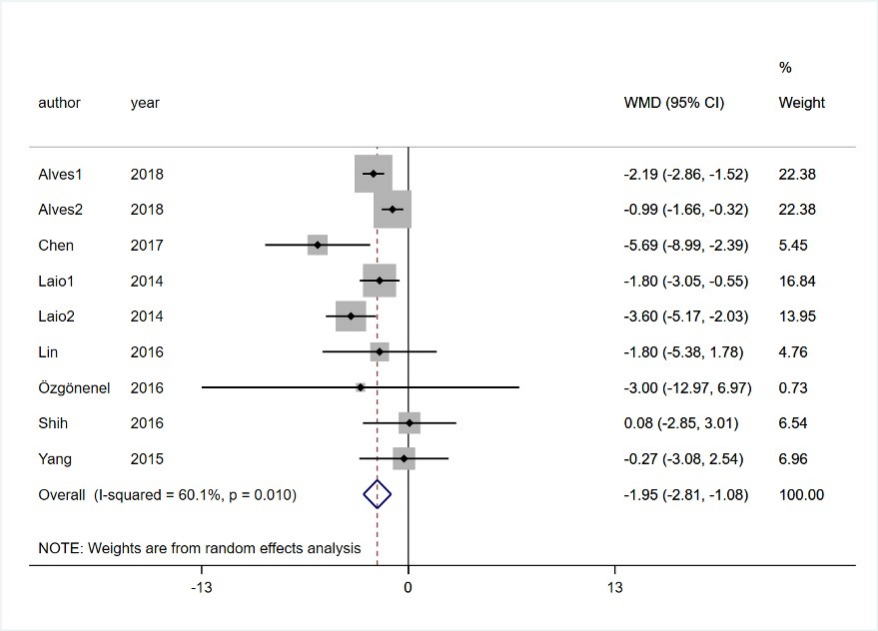
Forest plot of Mobility.

Seven studies [17, 19, 20, 22, 23, 29, 30] involving 237 participants with PD evaluated the effect of VR on mobility through TUGT. The shorter the test time, the stronger the mobility. We chose the random effect model because there was a high heterogeneity was observed (*I*^*2*^ = 60.1%, *P* = 0.001), pooled effect analysis showed that VR rehabilitation training had a significant effect on mobility (MD = -1.95, 95%CI = -2.81,-1.08, Z = 4.41, *P*<0.01, Fig 6).

### Results of secondary outcomes

#### Global motor function

**Fig 7:**
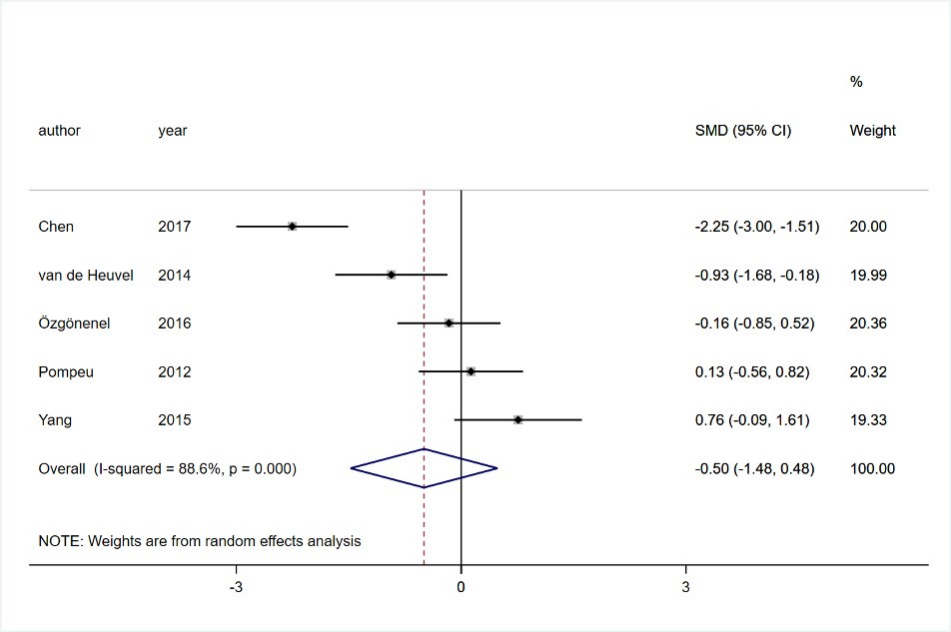
Forest plot of Global motor function.

Five studies [17, 20, 21, 25, 30] involving 164 participants with PD reported the effects of VR technology on global motor function. We chose the random effect model to pool effects owing to a significant heterogeneity was observed (*I*^2^ = 88.6%, *P*<0.01). There was no significant effect on global motor function between VR rehabilitation training and conventional training (SMD = -0.50, 95%CI = -1.48, 0.48, Z = 0.99, *P* = 0.32, Fig 7).

#### ADL

**Fig 8:**
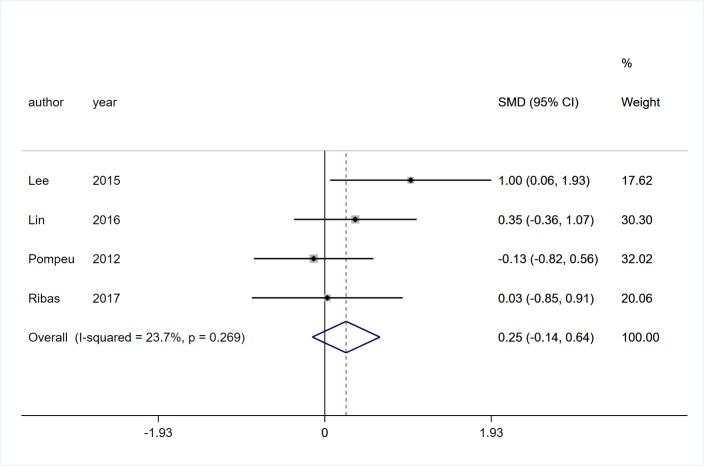
Forest plot of ADL.

Four studies [18, 21, 24, 29] involving 103 participants with PD reported the effects of VR technology on activities of daily life. Low heterogeneity was observed (*I*^*2*^ = 23.7%, *P* = 0.269). Pooled SMD presented that VR rehabilitation training had no significant effect on ADL (SMD = 0.25, 95%CI = -0.14, 0.64, Z = 1.24, P = 0.216, Fig 8).

#### Quality of life

**Fig 9:**
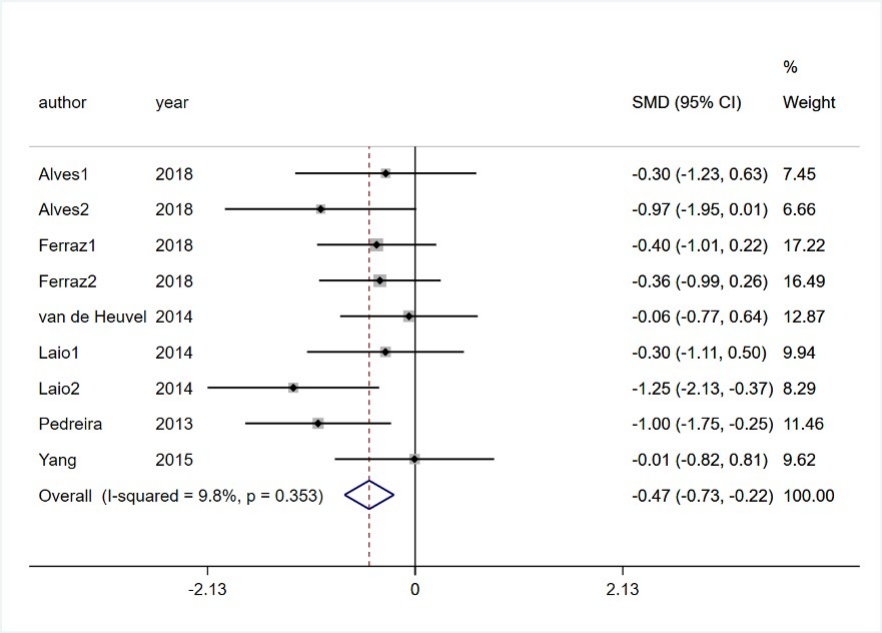
Forest plot of Quality of life.

Six studies [17, 19, 22, 25, 27, 31] involving 248 participants with PD reported the effects of VR technology on the changes of patients’ quality of life. Statistical heterogeneity was very low (*I*^2^ = 9.8%, *P* = 0.353). Pooled SMD showed that VR rehabilitation training was better in improving the quality of life of patients with PD (SMD = -0.47, 95%CI = -0.73, -0.22, Z = 3.64, *P*<0.01, Fig 9).

#### Perceived confidence in balance

**Fig 10:**
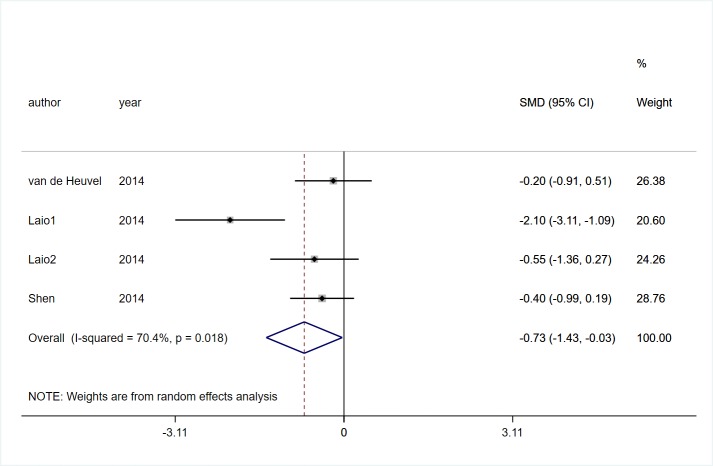
Forest plot of Perceived confidence in balance.

Three studies [22, 25, 26] involving 104 participants with PD reported the effects of VR technology on perceived confidence in balance. A significant heterogeneity was observed (*I*^2^ = 70.4%, *P* = 0.018). Pooled SMD presented that VR rehabilitation training had a significant effect on perceived confidence in balance than control group (SMD = -0.73, 95%CI = -1.43, -0.03, Z = 2.05, *P* = 0.040, Fig 10).

#### Neuropsychiatric symptoms

**Fig 11:**
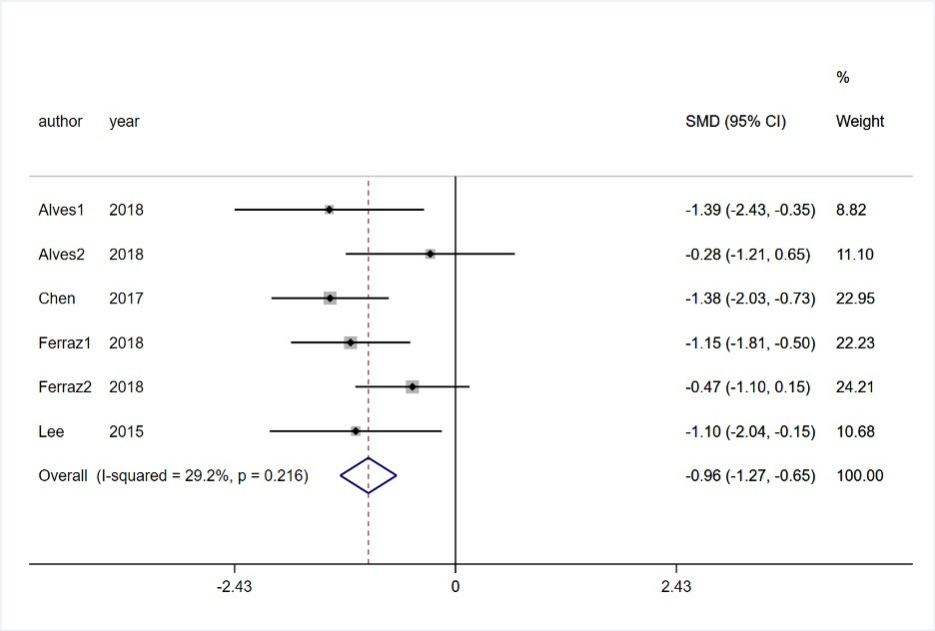
Forest plot of Neuropsychiatric symptoms.

Four studies [19, 20, 24, 27] involving 184 participants with PD reported the effect of VR on neuropsychiatric symptoms which were recorded by BAI, BDI and HAMD. There was a low statistical heterogeneity between studies (*I*^2^ = 29.2%, *P* = 0.216).Pooled SMD showed that VR rehabilitation training had a significant positive effect than control group (SMD = -0.96, 95%CI = -1.27, -0.65, Z = 6.07, *P*<0.01, Fig 11).

#### Cognitive function

**Fig 12:**
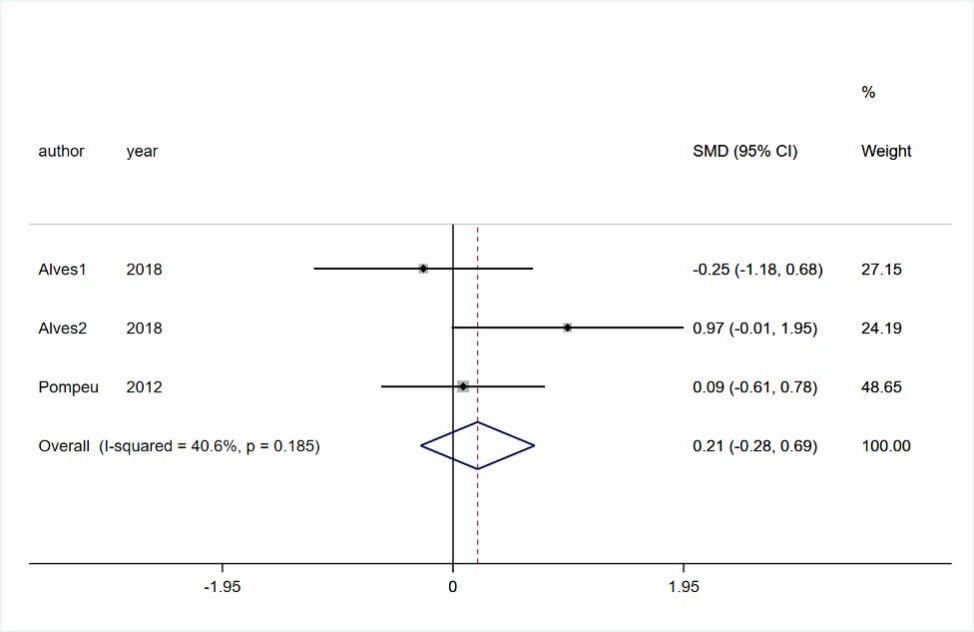
Forest plot of Cognitive function.

Only two studies [19, 21] involving 68 participants with PD study provided complete data on cognitive function changes, and there was no significant difference in cognitive function between two groups (SMD = 0.21, 95%CI = -0.28, 0.69, Z = 0.84, *P* = 0.399, Fig 12). Statistical heterogeneity remained low (*I*^2^ = 40.6%, *P* = 0.185).

#### Adverse events

Eight studies [21, 22, 24–26, 28, 29, 32] mentioned that adverse events occurred during the intervention would be observed. However, only one trial [29] reported that four patients developed mild dizziness and one patient developed severe dizziness and vomiting during the intervention.

### Publication bias

**Fig 13:**
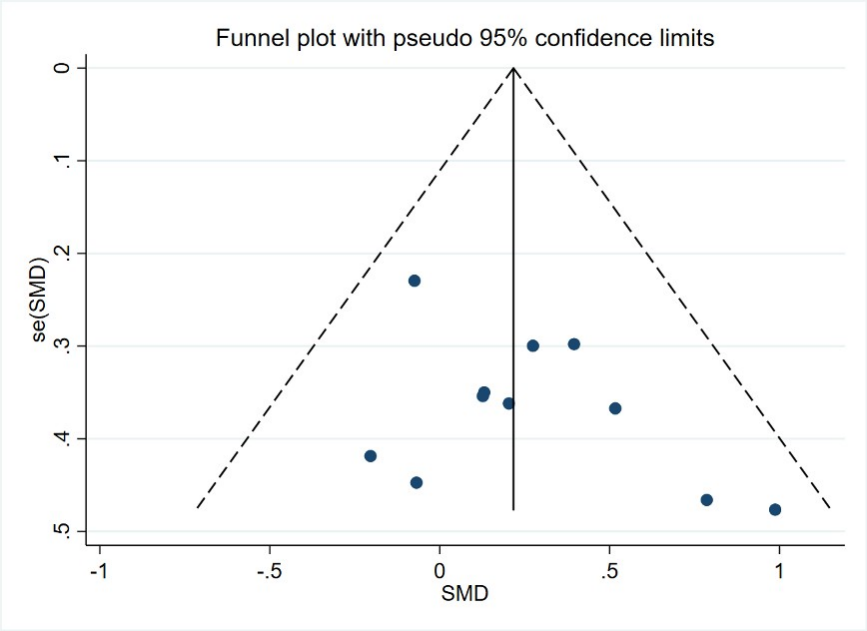
Funnel plot.

Finally, a funnel plot was constructed to identify any judge the publication bias. Since it included only the number of articles with balance function greater than 10 were included in the meta-analysis was greater than 10, we made funnel plot for it (Fig 13). The plot showed that the effects of each trial had relatively uniform distribution on both sides of the bottom. However due to the sample size of each article included in this research was small, and the rest of the outcomes were not suitable for making funnel plot, the existence of publication bias cannot be ruled out.

## Discussion

A total of 555 patients with PD in 16 studies were included in this systematic review. The results suggested that rehabilitation training based on VR technology is likely more effective than conventional training in improving PD patients' balance function mobility, step and stride length, quality of life and neuropsychiatric symptoms. Therefore, we believe that VR technology can at least be regarded as a rehabilitation therapy as effective as traditional training, and to some extent better than it. However, the results of this study should be treated with caution due to differences in treatment intensity, intervention duration, and effect size among the included studies.

VR is a new technology emerging in recent years, which is rapidly gaining popularity as a PD treatment tool due to its novelty in training methods and its ability to provide personalized rehabilitation training [33].Various professional technology platforms and treatment schemes based on VR are also under active development and research, and the number of researches on the application of VR in the field of neural rehabilitation is surging [34–36]. *Sampson et al*. found that the application of computer-based virtual robot training combined with functional electrical stimulation can improve the feasibility of arm movement in multiple patients and reduce the injury to the proximal arm, and patients have excellent compliance to intervention without side effects [37]. *Mahajan et al*. carried out relevant studies on driving operation training based on VR system, and found that this training method can effectively improve the position sensing ability of patients and can be an ideal choice for rehabilitation training of patients with tremor symptoms [38]. People with PD are unable to respond to changing conditions [39]. VR technology in PD is deepening, but the relevant conclusions are still controversial. Our systematic literature review findings are consistent with previous systematic reviews on stroke and Alzheimer's patients [40, 41], suggesting that VR intervention is superior to conventional rehabilitation training in improving the balance function and other indicators of the subjects.

Some included applied VR technology to balance training, dance training and other rehabilitation training, some studies used somatosensory game software to train patients, and some studies applied VR technology to rehabilitation training on the basis of routine exercise. The training time varied from 30 to 60 minutes for each session. In terms of training frequency, some studies conducted 2–3 times per week, while others conducted 5 times per week, and the total training time varied from 4 weeks to 12 weeks. In addition, VR technology equipment used in each study is not the same, and the conventional physical rehabilitation mode implemented by the control group in each study is not quite the same, which may be the reason for heterogeneity of results. Currently, there is no research that has shown which form of VR technology training or which treatment intensity has the best effect. It was pointed that PD patients have different tremor amplitude and rhythm patterns in different clinical stages (Hoehn-Yahr scale), which may be related to increased muscle tension and inactivity in patients with disease progression [42].As the Hoehn-Yahr stage of patients included in each study was different, the intervention effect will be affected to some extent, which may be one of the reasons for the heterogeneity of our study results. In future studies, targeted interventions for patients of different stages can be developed to eliminate such heterogeneity.

We analyzed VR's role in PD rehabilitation. Firstly, in the rehabilitation process, sports training based on VR technology can play a crucial role in improving the motor function of patients with nerve damage by reshaping the contra lateral sensor motor cortex. It can improve the ability of the brain to perceive, process and integrate information, so that patients can better maintain balance and control posture. Next, VR technology can generate instant feedback on a patient's audio-visual senses and proprioception, which enables patients to be placed in the environment through a variety of sensing devices, so as to improve the compliance of rehabilitation training and patients' enthusiasm [26]. Training tasks of different difficulty levels were set by accurately evaluating the functional level of the patients. When patients complete the training, they can not only feel the challenge but also reap the surprise of success, prompting them to continue the desire to "break the barrier", and finally achieve the purpose of improving the body function. Thirdly, active completion of assigned training tasks can enhance motor nerve plasticity and improve muscle control [43].

VR intervention means observed in the literature were diverse, but most of them are carried out in the form of immersive games. Such a training method enables patients to have a deeper interest and improve their subjective initiative, so as to actively complete various rehabilitation training, form a virtuous cycle to improve their functional level. Finally, the intervention in VR environment stimulated the subjects to generate autobiographical memory, improving their ability to recall familiar and unknown scenes [44]. Compared with traditional rehabilitation training, VR technology has the advantage that it can precisely provide more individualized rehabilitation training programs according to the disease characteristics of different patients, and can upload the training data on the internet in real-time, It also promotes interaction between patients and the health system through the data synchronization between different devices, so as to improve the rehabilitation effect.

There were some limitations in this study that should be noted. Although a detailed literature search had been done, it has to be admitted that there is still the possibility of study omission, such as minority language or gray literature. Secondly, due to the particularity of VR technology interventions, the blind method cannot be applied to patients in the experimental group, which may lead to the deviation of subjective data reported by patients in the result evaluation. It is indeed difficult to implement double-blind method, but it is still the best design for randomized controlled studies. In addition, the VR intervention forms, training time and dosage, training intensity and outcome evaluation indexes adopted in the included studies are also different. Therefore, there is some heterogeneity in data collection, which is also the common limitation in other systematic evaluations of VR intervention. Finally, the cost of VR rehabilitation training was only reported in one article [28], and the economic benefits of this type of training have not been compared with the control group. In future studies, the cost of different type rehabilitation training should be used as an indicator for observation.

## Conclusions

Although our results support the conclusion that VR is likely to be a more effective means of rehabilitation for PD patients, more rigorously designed, standardized interventions and larger sample size multicenter randomized controlled trials are needed to provide a stronger evidence-based basis to verify the potential advantages of VR.

## Supporting information

S1 Checklist (DOCX)

S1 Appendix (DOCX)
