# Supplementary material for: Effects of virtual reality rehabilitation training on gait and balance in patients with Parkinson's disease: A systematic review
Source: PLoS One. 2019 Nov 7;14(11):e0224819. doi: 10.1371/journal.pone.0224819 (PMC6837756; doi:10.1371/journal.pone.0224819)
Supplement: S1 Appendix — (DOCX) [file pone.0224819.s002.docx]

**S1 Appendix.**

**Search strategy: take the search process via Pubmed as an example**

#1 (Parkinson Disease[mh]) OR (Idiopathic Parkinson's Disease) OR (Lewy Body Parkinson Disease) OR (Lewy Body Parkinson's Disease) OR (Primary Parkinsonism) OR (Parkinsonism, Primary) OR (Parkinson Disease, Idiopathic) OR (Parkinson's Disease) OR (Parkinson's Disease, Idiopathic) OR (Parkinson's Disease, Lewy Body) OR (Idiopathic Parkinson Disease) OR (Paralysis Agitans) OR (Parkinsonian) OR (PD)

#2 (Virtual Reality Exposure Therapy[mesh]) OR (Virtual Reality Immersion Therapy) OR (Virtual Reality Therapy) OR (Reality Therapies, Virtual) OR (Reality Therapy, Virtual) OR (Therapies, Virtual Reality) OR (Therapy, Virtual Reality) OR (Virtual Reality Therapies) OR (x-box 360) OR (kinect) OR (wii) OR (VR)

#3 randomized controlled trial[pt]

#4 controlled clinical trial[pt]

#5 randomized [tiab]

#6 randomly[tiab]

#7 groups[tiab]

#8 #3 OR #4 OR #5 OR #6 OR #7

#9 #1 AND #2 AND #8
